# Case Report: PRaG 3.0 therapy for advanced cervical cancer with severe comorbidities

**DOI:** 10.3389/fonc.2026.1912098

**Published:** 2026-09-16

**Authors:** Xinyi Gu, Meiling Xu, Jiangwen Dai, Xia Li, Junjun Zhang, Pengfei Xing, Liyuan Zhang, Yuehong Kong

**Affiliations:** 1 Department of Oncology, Cancer Prevention and Treatment Institute of Chengdu, Chengdu Fifth People’s Hospital (The Second Clinical Medical College, Affiliated Fifth People’s Hospital of Chengdu University of Traditional Chinese Medicine), Chengdu, Sichuan, China; 2 PD-1 inhibitor, Radiotherapy, and Granulocyte-Macrophage Colony-Stimulating Factor (PRaG) Center, Center for Cancer Diagnosis and Treatment, The Second Affiliated Hospital of Soochow University, Suzhou, Jiangsu, China

**Keywords:** case report, cervical cancer, disitamab vedotin, PD-L1 inhibitor, PRaG 3.0, renal dysfunction, severe anemia, stereotactic body radiotherapy

## Abstract

Patients with advanced cervical cancer complicated by renal dysfunction and severe anemia are frequently ineligible for standard platinum-based chemotherapy, creating an urgent need for effective non-platinum salvage strategies. We report a patient with FIGO stage IVB poorly differentiated cervical squamous cell carcinoma with HER2 immunohistochemistry (IHC) 2+ expression, acute kidney injury caused by malignant ureteral obstruction (serum creatinine, 793 μmol/L), and severe anemia (hemoglobin, 47 g/L) at presentation. After bilateral nephrostomy, the patient received PRaG 3.0, a five-component regimen comprising stereotactic body radiotherapy, adebrelimab (anti-PD-L1), disitamab vedotin (HER2-directed antibody-drug conjugate), granulocyte-macrophage colony-stimulating factor, and interleukin-2. After two cycles, both the cervical primary lesion and pulmonary metastases showed marked regression, and the best overall response was partial response. Treatment was generally manageable; the only grade 3 treatment-related adverse event was an interleukin-2-associated generalized rash, which resolved after drug discontinuation and supportive care. At the last follow-up, progression-free survival exceeded 8 months, and both nephrostomy tubes had been removed. This case suggests that PRaG 3.0 may provide clinically meaningful disease control in selected patients with advanced cervical cancer and severe comorbidities who cannot tolerate platinum-based chemotherapy. Given the single-patient nature of this report, prospective validation is required.

## Introduction

Cervical cancer remains a major global health burden, particularly in regions with limited medical resources, where both incidence and mortality remain substantial (1). According to the 2022 estimates from the International Agency for Research on Cancer, approximately 661,021 new cases and 348,189 deaths occurred worldwide, ranking cervical cancer eighth in incidence and ninth in cancer-related mortality among all malignancies (2). In China, approximately 112,500 new cases and 55,700 deaths were reported in 2022 (3).

Patients with advanced cervical cancer commonly develop life-threatening complications, including renal failure, venous thromboembolism, and severe hemorrhage (4–6). Ureteral obstruction is particularly frequent in patients with FIGO stage IIIB disease or higher and may result from tumor compression or direct parametrial invasion (7). Ureteral obstruction and renal failure are clinically important adverse prognostic factors (7, 8). Mishra et al. (9) reported a 5-year survival rate of 47% in patients without ureteral obstruction, compared with 29% in those with unilateral or bilateral hydronephrosis; notably, no patient with concomitant renal failure survived beyond 18 months.

Anemia is also highly prevalent in cervical cancer and is positively associated with disease stage. In advanced-stage disease (FIGO IIB-IV), the prevalence can exceed 60%-80% (10). Major contributors include chronic tumor-related bleeding, malnutrition, inflammation, and treatment-related myelosuppression (11). Importantly, anemia is not merely a concomitant symptom: it can aggravate tumor hypoxia, reduce radiosensitivity and chemosensitivity, and limit the delivery of adequate systemic therapy, thereby compromising treatment efficacy (12, 13).

Current national and international treatment paradigms recommend platinum-based chemotherapy combined with immunotherapy, with or without antiangiogenic therapy, for metastatic cervical cancer (14, 15). However, cisplatin is contraindicated in patients with significant renal dysfunction, and carboplatin may also be unsuitable when renal function is severely compromised (16).The patient presented with active vaginal bleeding, and bevacizumab substantially increases the risk of hemorrhage-related adverse events (17). Monotherapy with immune checkpoint inhibitors yields limited anti-tumor efficacy, and clinical benefits are highly dependent on tumor PD-L1 expression levels (18).

Therefore, safe and effective salvage strategies are needed for patients with advanced cervical cancer and severe comorbidities who are not candidates for platinum-based chemotherapy. The PRaG 3.0 regimen consists of radiotherapy, PD-1/PD-L1 inhibitors, HER2 antibody-drug conjugate (ADC), GM-CSF and IL-2, which is supported by robust biological evidence. Radiotherapy combined with disitamab vedotin can induce immunogenic cell death in tumor cells and promote the release of tumor antigens (19). GM-CSF facilitates the maturation of dendritic cells and improves antigen presentation capacity. Anti-PD-1/PD-L1 inhibitors relieve the immunosuppressive state of T cells and activate anti-tumor immune responses (20). Low-dose IL-2 further expands functional effector T cells and memory T cells (21). This regimen sequentially realizes tumor antigen release, antigen presentation, immune checkpoint blockade and effector T cell maintenance. It amplifies systemic anti-tumor immune responses through multi-link synergism and promotes the occurrence of the abscopal effect. We report a patient with advanced cervical squamous cell carcinoma accompanied by severe anemia and renal dysfunction who received PRaG 3.0. The patient achieved sustained tumor regression, a best response of partial response, removal of bilateral nephrostomy tubes, and progression-free survival exceeding 8 months at the last follow-up.

## Case presentation

The patient is a 57-year-old female with no prior history of hypertension, diabetes mellitus, or chronic kidney disease. In October 2025, she presented with irregular vaginal bleeding. PET-CT revealed a large uterine soft-tissue mass measuring 10.8 × 9.9 cm with invasion of the bladder and vagina, multiple pulmonary metastases (largest diameter, 5.6 cm), and extensive nodal metastases involving the supraclavicular, mediastinal, abdominal, retroperitoneal, pelvic, and right inguinal regions (**Figures 1A–D**). Cervical biopsy confirmed poorly differentiated squamous cell carcinoma. Immunohistochemistry showed PD-L1 positivity (combined positive score, 25), HER2 IHC 2+, Trop-2 3+ (100%), MLH1(+), PMS2(+), MSH2(+), MSH6(+), AE1/AE3(+), CK7(+), p16(+), CK5/6(partial +), p63(+), p40(scattered +), Ki-67 80%, ER(partial +), PR(-), PAX8(-), Syn(-), and CD56(-). The disease was staged as FIGO 2018 IVB.

**Figure 1:**
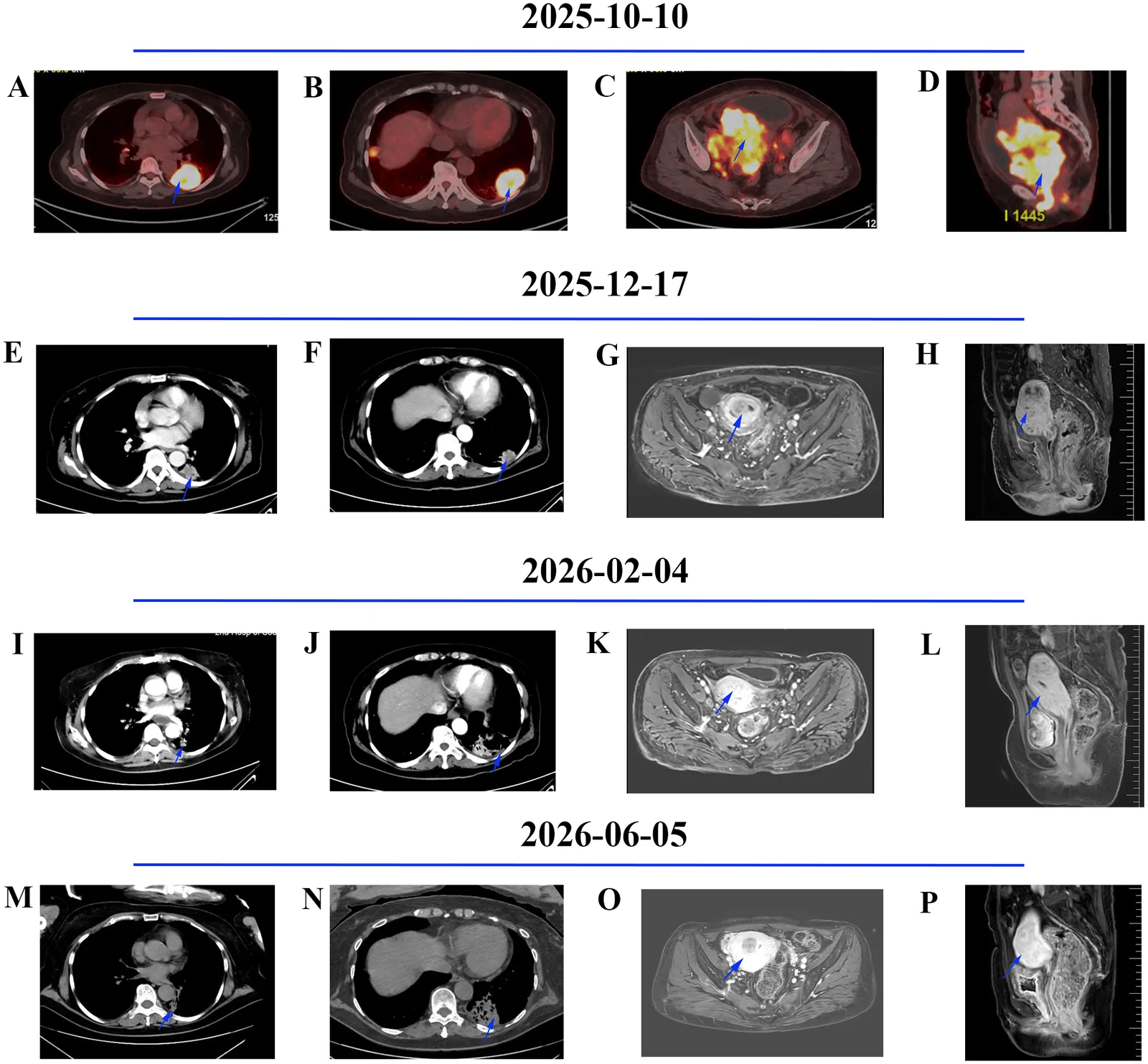
Imaging assessment at baseline **(A–D)**, after cycle 2 **(E–H)**, after cycle 4 **(I–L)**, and after cycle 9 **(M–P)**.

At admission, the serum creatinine level was 793 μmol/L(KDIGO AKI stage 3), consistent with acute kidney injury secondary to malignant ureteral obstruction. Bilateral nephrostomy was performed from October 10 to October 14, 2025, after which serum creatinine decreased to 239 μmol/L(KDIGO AKI stage 2). Meanwhile, due to vaginal bleeding, the patient’s hemoglobin level dropped to 47 g/L (CRA grade 3). We transfused 5 units of suspended red blood cells, and her hemoglobin level increased to 72 g/L (CRA grade 3). Given her compromised renal function, severe anemia, and Eastern Cooperative Oncology Group performance status of 2, a multidisciplinary team comprising gynecologic oncology, radiation oncology, medical oncology, urology, pathology, and radiology considered PRaG 3.0 appropriate for rapid tumor control and hemostasis; before receiving any protocol treatment, the patient was prospectively enrolled in the phase II PRaG 3.0 trial (NCT05115500; protocol version 1.4; Ethics Committee of the Second Affiliated Hospital of Soochow University, approval No. JD-LK-2021121-A03, approved September 11, 2025) and provided trial-specific written informed consent.

## Therapeutic intervention, follow-up, and outcomes

The regimen consisted of an induction phase (phase A, four cycles) followed by consolidation/maintenance treatment (phase B), with treatment repeated every 3 weeks (**Figure 2**).

**Figure 2:**
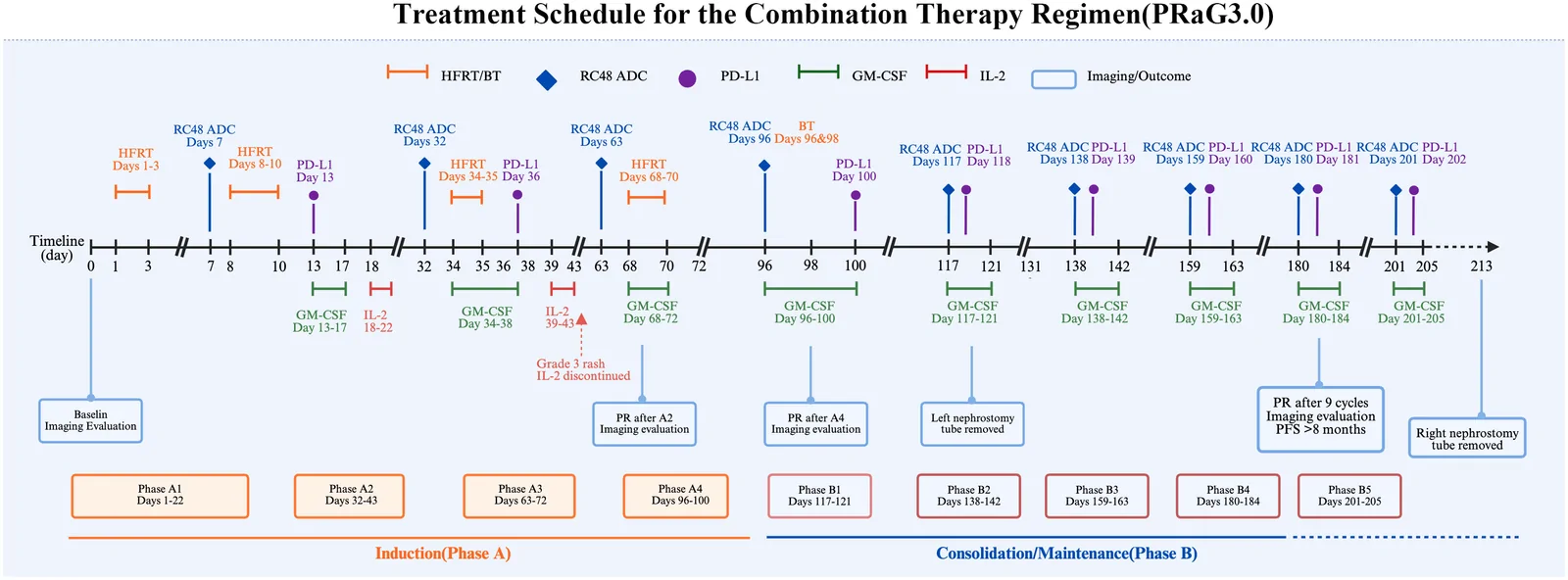
Timeline of the patient’s clinical course and PRaG 3.0 treatment process.

Phase A1 (October 17-29, 2025): cervical lesion HFRT, 24 Gy in 3 fractions (October 17-19); disitamab vedotin, 120 mg (October 23); left lower-lobe lung metastasis HFRT, 24 Gy in 3 fractions (October 24-26); adebrelimab, 1200 mg (October 29); GM-CSF, 200 μg/day for 5 days (October 29-November 2); and IL-2, 200 IU/day for 5 days (November 3-7).

Phase A2 (November 17-28, 2025): disitamab vedotin, 120 mg; cervical HFRT, 10 Gy in 2 fractions (November 19-20); adebrelimab, 1200 mg; GM-CSF, 200 μg/day for 5 days (November 19-23); and IL-2, 200 IU/day for 5 days (November 24-28).

After two cycles, MRI and CT showed reduction of the cervical tumor to 5.8 × 5.6 cm and the pulmonary metastasis to 3.3 cm, consistent with partial response (**Figures 1E–H**).

Phase A3 (December 18-27, 2025): disitamab vedotin, 120 mg; left lower-lobe HFRT, 24 Gy in 3 fractions (December 23-25); adebrelimab, 1200 mg; and GM-CSF, 200 μg/day for 5 days (December 23-27). IL-2 was permanently discontinued because of a grade 3 generalized rash during the preceding cycle.

Phase A4 (January 20-24, 2026): disitamab vedotin, 120 mg; brachytherapy to the high-risk clinical target volume (5.51 Gy on January 20 and 4.53 Gy on January 22); adebrelimab, 1200 mg; and GM-CSF, 200 μg/day for 5 days (January 20-24).

After four cycles, MRI/CT showed further disease control, with the cervical tumor measuring 4.9 × 4.1 cm and the lung metastasis measuring 2.4 cm, maintaining partial response (**Figures 1I–L**). Bilateral hydronephrosis improved, and the left nephrostomy tube was removed on February 24, 2026.

Consolidation/maintenance treatment (Phase B1-B5, February 10-May 17, 2026) consisted of disitamab vedotin 120 mg plus adebrelimab 1200 mg, with GM-CSF 200 μg/day for 5 days in each cycle. Tumor markers and hematologic and renal parameters remained clinically stable during treatment (**Figures 3A–D**).

**Figure 3:**
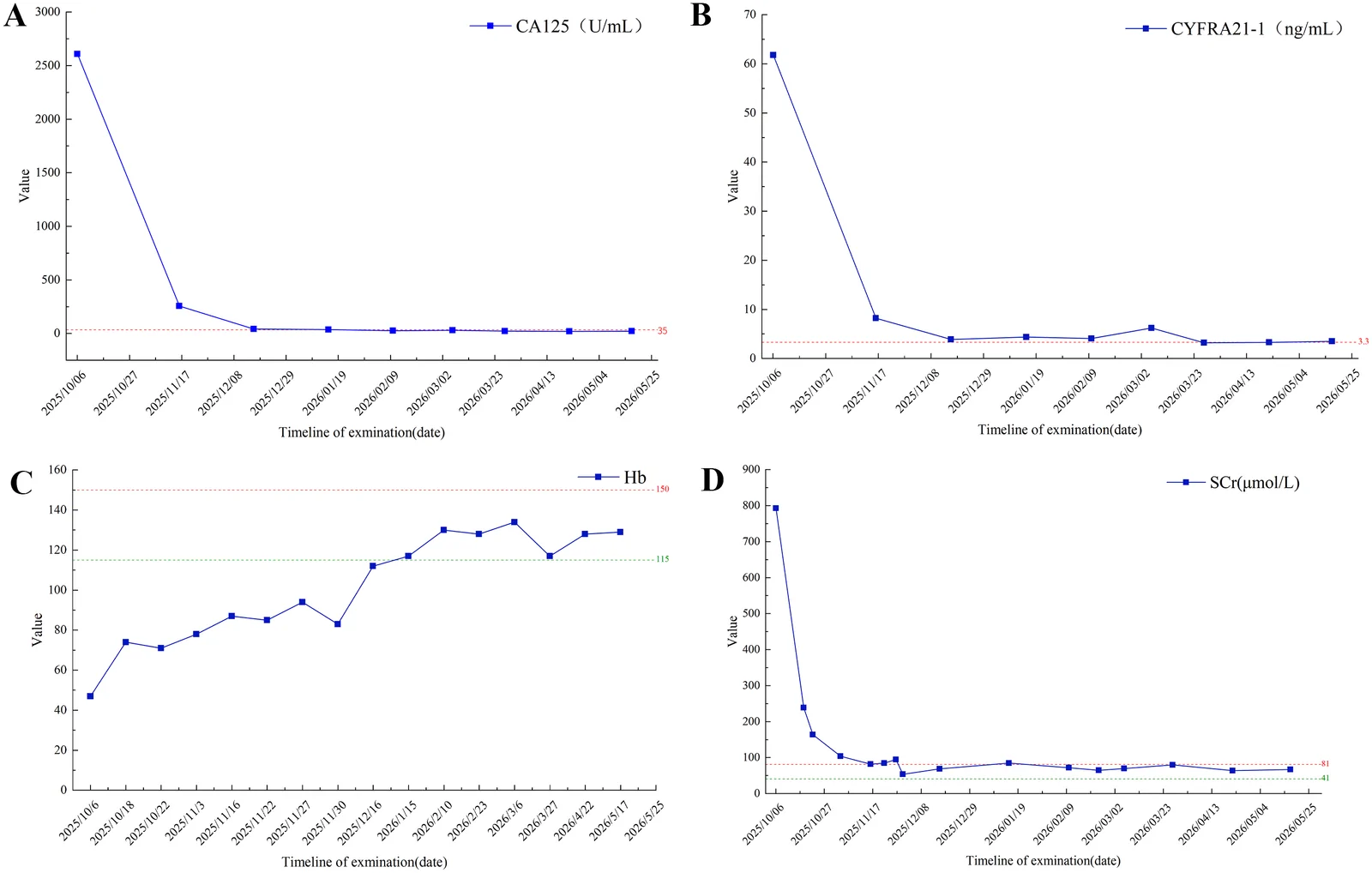
Tumor markers, hematologic and renal parameters at baseline and during treatment. **(A, B)** CA125 and CYFRA 21-1; **(C, D)** hemoglobin and serum creatinine.

After nine cycles, MRI/CT showed a cervical tumor measuring 4.9 × 4.1 cm and a pulmonary metastasis measuring 1.8 cm, maintaining partial response (**Figures 1M–P**). The right nephrostomy tube was removed on June 10, 2026. The only grade ≥3 treatment-related adverse event was an IL-2-associated grade 3 rash, which resolved after IL-2 discontinuation and supportive care. No other grade 3–4 toxicities were observed. At the last follow-up, progression-free survival exceeded 8 months.

## Discussion

PRaG therapy, first proposed in 2019, is an immunotherapy-based treatment paradigm consisting of a PD-1/PD-L1 inhibitor (P), radiotherapy (Ra), and granulocyte-macrophage colony-stimulating factor (GM-CSF; G). Mechanistically, radiotherapy may induce immunogenic cell death and release tumor-associated antigens, thereby generating an *in situ* vaccine-like effect. GM-CSF can promote dendritic-cell recruitment and maturation, enhancing antigen uptake, processing, and presentation. PD-1/PD-L1 blockade may then restore exhausted T-cell activity and facilitate antitumor immune responses (20, 22). In a previous open-label phase II study involving 54 patients with refractory metastatic solid tumors who had progressed after standard therapies, PRaG achieved an objective response rate (ORR) of 16.7%, a disease control rate (DCR) of 46.3%, median progression-free survival (mPFS) of 4.0 months, and median overall survival (mOS) of 10.5 months (20). For patients with HER2 IHC-positive solid tumors, PRaG 3.0 was subsequently developed by incorporating a HER2-targeted antibody-drug conjugate and IL-2 into the PRaG backbone. Interim data from the ongoing study (NCT05115500) showed an ORR of 41.4% and a DCR of 72.4% among 29 patients with HER2-positive solid tumors, including two patients who achieved minimal residual disease-negative complete remission (23). The present case provides clinical support for evaluating this intensified regimen in cervical cancer patients with severe comorbidities, although causal inference cannot be made from a single case.

At presentation, the patient had active vaginal bleeding and a large cervical mass. Hypofractionated radiotherapy was selected to achieve rapid hemostasis and local tumor control. Beyond cytoreduction, radiotherapy may facilitate antigen release, increase major histocompatibility complex expression, promote dendritic-cell priming, and reshape the tumor microenvironment, thereby providing a mechanistic rationale for combining radiotherapy with immune checkpoint blockade (24). Subsequent irradiation selectively targeted a limited number of lesions to promote antigen release and enhance local control, consistent with the multi-site RT framework described by Patel et al. (25)and Chang et al (26). Disease-specific clinical evidence in cervical cancer has also emerged, mainly in the definitive chemoradiotherapy setting for locally advanced disease. In the phase III KEYNOTE-A18 trial, pembrolizumab added to concurrent chemoradiotherapy and continued as maintenance therapy improved progression-free survival in patients with newly diagnosed, high-risk locally advanced cervical cancer (27). In contrast, the phase III CALLA trial did not show a statistically significant progression-free survival benefit when durvalumab was added to definitive chemoradiotherapy for unselected locally advanced cervical cancer (28). Early-phase studies, including atezolizumab administered before and/or during chemoradiotherapy for locally advanced cervical cancer, further support the feasibility of integrating immune checkpoint inhibition with radiation-based treatment, although optimal sequencing, patient selection, and biomarkers remain unresolved (29). Taken together, these data support the biological and clinical rationale for radiation-immunotherapy integration in cervical cancer, while also indicating that benefit depends on disease setting, checkpoint inhibitor, and patient selection.

For recurrent or metastatic cervical cancer, the strongest evidence for immunotherapy currently comes from combinations with systemic therapy rather than concurrent chemoradiotherapy. KEYNOTE-826 demonstrated that pembrolizumab plus chemotherapy improved survival outcomes compared with chemotherapy alone in patients with persistent, recurrent, or metastatic cervical cancer whose tumors expressed PD-L1 (14). The phase III COMPASSION-16 study further showed that cadonilimab plus platinum-based chemotherapy, with or without bevacizumab, improved mPFS compared with chemotherapy alone in treatment-naive recurrent or metastatic cervical cancer (12.7 vs. 8.1 months; HR, 0.62; P < 0.0001) (15). These studies establish immune checkpoint blockade as a key component of first-line systemic therapy in advanced cervical cancer. However, the patient in this report was not an appropriate candidate for standard platinum-based therapy because of severe renal dysfunction and anemia. Adebrelimab, a humanized anti-PD-L1 IgG4 monoclonal antibody, was therefore selected as the immune checkpoint inhibitor component on the basis of the patient’s PD-L1-positive tumor status and the need for a platinum-free regimen with acceptable tolerability in the setting of major comorbidities (30, 31).

To further enhance antitumor activity, disitamab vedotin (RC48) and IL-2 were incorporated into the PRaG backbone. Disitamab vedotin is a HER2-directed antibody-drug conjugate that has demonstrated activity in HER2-expressing solid tumors, including gastric and urothelial cancers (32–34). Emerging data also suggest antitumor activity of disitamab vedotin-based strategies in recurrent or metastatic cervical cancer and other gynecologic malignancies with HER2 expression (35, 36). In the present case, HER2 IHC 2+ expression provided a rational basis for including disitamab vedotin. The observed regression of both irradiated and non-irradiated lesions is clinically notable, but the relative contribution of radiotherapy, immune checkpoint blockade, disitamab vedotin, GM-CSF, and IL-2 cannot be separated in a single-patient report.

IL-2 can expand natural killer cells and effector T-cell populations and may therefore complement PD-1/PD-L1 blockade (37). In this patient, however, IL-2 was discontinued after a grade 3 generalized rash during the second treatment cycle. Immune-related cutaneous adverse events have been associated with improved tumor control in some immunotherapy studies (38), but this association should be interpreted cautiously and cannot be used as a surrogate efficacy marker in an individual patient. Newer PD-1/IL-2 bifunctional agents, including IBI363 and AWT020, are under clinical investigation and may offer more selective IL-2 pathway modulation (39, 40). The inclusion of IL-2 in PRaG 3.0 is therefore biologically plausible, but its optimal dose, schedule, patient selection, and safety management require further study.

Serial monitoring of peripheral blood lymphocyte subsets was performed to explore immune dynamics during treatment. After the first treatment cycle, the proportions of activated CD4+ central memory T cells(activated CD4+Tcm), activated CD8+ central memory T cells(activated CD8+Tcm), and activated CD8+ effector memory T cells(activated CD8+Tem) increased markedly and then gradually declined during subsequent cycles (**Figures 4A–C**). CD4+ Tcm express CD62L and CCR7, which mediate their homing to secondary lymphoid organs. Upon re-stimulation with specific antigens, these cells rapidly proliferate and differentiate into effector CD4+T cells. By secreting cytokines such as IL-2 and IFN-γ, they regulate the long-term maintenance and formation of memory reservoirs of CD8+Tcm, facilitate the activation and cytotoxicity of CD8+Tem, support the expansion and functional maturation of both subsets of CD8^+^ memory T cells, and synergistically establish a comprehensive anti-tumor memory immune response (41–43).Prior studies have linked expansion of selected memory and activated T-cell subsets with favorable outcomes after immune checkpoint blockade and other immunotherapies (44–46). Takeuchi et al. (45) demonstrated that elevated peripheral blood CD4+Tcm could predict the clinical benefit of PD-1 inhibitor therapy in patients with malignant melanoma, and CD4+Tcm with the CD27+Fas–CD45RACCR7+ phenotype were markedly enriched in long-term survivors. Another study enrolling patients with stage IV malignant melanoma receiving PD-1 inhibitor treatment revealed that treatment responders maintained higher levels of CD8+Tem before and after the first treatment cycle compared with non-responders (47).In this case, the transient expansion followed by contraction may reflect decreasing antigenic stimulation after tumor regression or a physiological contraction phase of the adaptive immune response (48). However, peripheral immune monitoring remains exploratory; these findings should be considered hypothesis-generating rather than definitive evidence of durable antitumor immune memory.

**Figure 4:**
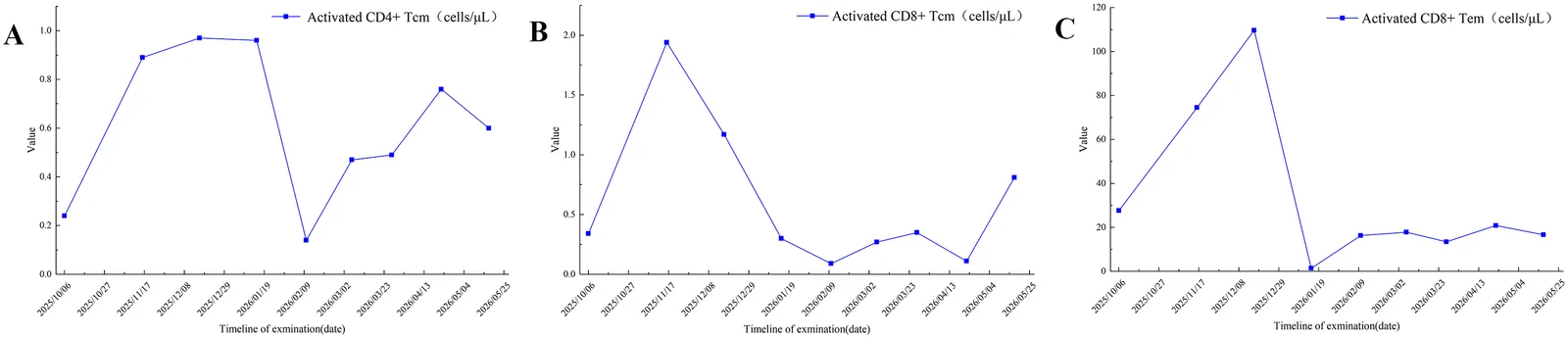
Proportions of peripheral T lymphocyte subsets at baseline and after each treatment cycle. **(A)** Activated CD4^+^ central_memory T cells (Activated CD4^+^ Tcm cells); **(B)** Activated CD8^+^ central_memory T cells (Activated CD8^+^ Tcm cells); **(C)** Activated CD8^+^ effector_memory T cells (Activated CD8^+^ Tem cells).

## Conclusion

This case describes the successful use of PRaG 3.0 in a patient with FIGO IVB cervical squamous cell carcinoma complicated by acute kidney injury from malignant ureteral obstruction and severe anemia, conditions that limited the feasibility of platinum-based chemotherapy. PRaG 3.0 was associated with rapid tumor regression, removal of bilateral nephrostomy tubes, progression-free survival exceeding 8 months, and manageable toxicity. For carefully selected patients with advanced cervical cancer and severe comorbidities, this platinum-free immunotherapy-based combination may represent a potential salvage strategy. Nevertheless, the findings are limited by the single-case design, concurrent use of multiple therapeutic components, and lack of long-term survival data. Prospective studies, including the ongoing phase II trial of PRaG 3.0 (NCT05115500), are required to define efficacy, safety, biomarkers, and optimal patient selection.

## Data Availability

The original contributions presented in the study are included in the article/supplementary material. Further inquiries can be directed to the corresponding authors.
